# Prevention of Yellow Fever

**Published:** 1889-07

**Authors:** 


PREVENTION OF YELLOW FEVER in Florida and the South.—A paper read before the Baltimore Academy of Medicine, Dec., 1888, by W. C. VanBibber, A. M., M. D., member of the Medical and Chirurgical Faculty of Maryland, member of the American Public Health Association, etc., Baltimcre Md., 1889.

The paper is quite interesting and contains valuable suggestions to the question. If a city, town or locality kept *continuously* clean, free from all " ," will yellow fever become epidemic? The author replies, "*no, a place may be made and kept yellow fever proof.*

